# Platelet-rich plasma: why intra-articular? A systematic review of preclinical studies and clinical evidence on PRP for joint degeneration

**DOI:** 10.1007/s00167-013-2743-1

**Published:** 2013-11-26

**Authors:** G. Filardo, E. Kon, A. Roffi, B. Di Matteo, M. L. Merli, M. Marcacci

**Affiliations:** 1 Nano-Biotechnology Laboratory, II Orthopaedic Clinic, Rizzoli Orthopaedic Insitute, Via di Barbiano n. 1/10, 40136 Bologna, Italy; 2 Biomechanics Laboratory, II Orthopaedic Clinic, Rizzoli Orthopaedic Insitute, Via di Barbiano n. 1/10, 40136 Bologna, Italy

**Keywords:** PRP, Growth factors, Knee, Intra-articular, Injection, Cartilage

## Abstract

**Purpose:**

The aim of this review was to analyze the available evidence on the clinical application of this biological approach for the injective treatment of cartilage lesions and joint degeneration, together with preclinical studies to support the rationale for the use of platelet concentrates, to shed some light and give indications on what to treat and what to expect from intra-articular injections of platelet-rich plasma (PRP).

**Methods:**

All in vitro, in vivo preclinical and clinical studies on PRP injective treatment in the English language concerning the effect of PRP on cartilage, synovial tissue, menisci, and mesenchymal stem cells were considered. A systematic review on the PubMed database was performed using the following words: (platelet-rich plasma or PRP or platelet concentrate or platelet lysate or platelet supernatant) and (cartilage or chondrocytes or synoviocytes or menisci or mesenchymal stem cells).

**Results:**

Fifty-nine articles met the inclusion criteria: 26 were in vitro, 9 were in vivo, 2 were both in vivo and in vitro, and 22 were clinical studies. The analysis showed an increasing number of published studies over time. Preclinical evidence supports the use of PRP injections that might promote a favourable environment for joint tissues healing. Only a few high-quality clinical trials have been published, which showed a clinical improvement limited over time and mainly documented in younger patients not affected by advanced knee degeneration.

**Conclusions:**

Besides the limits and sometimes controversial findings, the preclinical literature shows an overall support toward this PRP application. An intra-articular injection does not just target cartilage; instead, PRP might influence the entire joint environment, leading to a short-term clinical improvement. Many biological variables might influence the clinical outcome and have to be studied to optimize PRP injective treatment of cartilage degeneration and osteoarthritis.

**Level of evidence:**

IV.

## Introduction

A healthy joint requires a fine-tuned balance between molecular signals regulating homeostasis, damage, restoration, and remodelling. This balance is determined both at the level of single cells and the whole tissue architecture, and it also involves interactions among different tissues such as cartilage, bone, synovium, ligaments, tendons, and menisci [46]. Different factors are able to impair the maintenance of homeostasis in a joint that has been damaged or strained, and they may progressively lead to osteoarthritis (OA) [27, 29].

A wide spectrum of treatments is available, from non-pharmacological modalities to dietary supplements and pharmacological therapies, as well as minimally invasive procedures involving injections of various substances aimed at restoring joint homeostasis and providing clinical improvement and, possibly, a disease-modifying effect [39]. When these treatments fail, more invasive surgical approaches can be attempted to avoid metal resurfacing through the restoration of the mechanical balance and the regeneration of the articular surface, although results are still controversial [21, 22]. Even though some of these approaches have been shown to offer a satisfactory clinical outcome at midterm follow-up, rehabilitation is long and results are often unpredictable, incomplete, and limited over time [10, 15, 16, 18, 37].

The search for a minimally invasive solution to improve the status of the joint surface and allow a fast return to full activity is therefore highly desirable. In this landscape, a novel promising injective treatment is platelet-rich plasma (PRP), a blood derivative that has a higher platelet concentrate than whole blood. When activated, platelets release a group of biologically active proteins that bind to the transmembrane receptors of their target cells, thus leading to the expression of gene sequences that ultimately promote cellular recruitment, growth, and morphogenesis, and modulating inflammation as well [3]. Therefore, PRP represents an appealing biological approach to favour the healing of tissues otherwise doomed by a low healing potential, such as cartilage. This led to the wide use of PRP, which shows promising results as a minimally invasive injective treatment of cartilage degeneration and OA, both in preclinical and clinical studies [40, 67]. However, besides the increasing interest both among physicians and the scientific community, results are sometimes contradictory with no clear treatment indications, due to low-level clinical studies and the lack of understanding on the mechanism of action of this blood derivative [40].

The aim of this review was to analyze systematically the available evidence on the clinical application of this biological approach for the injective treatment of cartilage lesions and joint degeneration, together with preclinical studies to support the rationale for this use of platelet concentrates, to shed some light and give indications on what to treat and what to expect from intra-articular injections of PRP.

## Materials and methods

All in vitro, in vivo preclinical and clinical studies on PRP injective treatment in the English language concerning the effect of PRP on cartilage, synovial tissue, and menisci were considered. Since PRP injections could be used as augmentation procedure after bone marrow stimulation techniques or other cell type transplantations, the analysis of studies dealing with the PRP effect on mesenchymal stem cells (MSCs) of various origins for cartilage treatment was also included. A systematic review on the PubMed database was performed using the following words: (Platelet-Rich Plasma OR PRP OR Platelet Concentrate OR Platelet Lysate OR Platelet Supernatant) AND (Cartilage OR Chondrocytes OR synoviocytes OR menisci OR mesenchymal stem cells). Reference lists from the selected papers were also screened. Relevant data were then extracted and collected in three tables, separating in vitro, in vivo preclinical studies, and clinical studies (case reports were not considered) (Tables 1, 2, 3). Two studies focused on in vitro and preclinical in vivo evaluations and were reported in both Tables 1 and 2. The in vitro studies were divided according to the cell population targeted. With regard to clinical trials, only comparative and randomized controlled trials (RCTs) were discussed further in the present manuscript.

**Table 1:** In vitro studies

Publications	PRP characteristics	PRP effects
Chondrocytes		
Yin [77]	Platelet count: 2,604 ± 602 × 10^3^/ml Activation: – No leukocytes	Increase in proliferation and ECM deposition in the integration area between agarose scaffold and cartilage samples Higher scaffold integration strength
Muraglia [54]	Platelet count: 10 × 10^6^/μl No activation Leukocytes: –	Increase in cell proliferation more than FCS, also in chondrocytes from elderly patients
Hildner [28]	Platelet count: – Activation: – Leukocytes: –	Increase in proliferation Better redifferentiation potential than FCS expanded cells
Park [57]	Platelet count: 6–10 × 10^6^/μl No activation Leukocytes: –	Dose-dependent increase in chondrocytes proliferation maintained at 4 days in 5, 10, 20 % PRP Chondrogenic phenotype maintenance Time-dependent increase in angiogenic and antiangiogenic factors expression (VEGF, ChM-I)
Lee [44]	Platelet count: – Activation: – No leukocytes	Increased chondrocyte proliferation in time-dependent manner Enhanced hydrogel scaffold–chondrocyte maturation Immediate increase in CB1 and CB2 mRNA expression
Pereira [59]	Platelet count: 1 × 10^7^/ml Activation: freezing and thawing Leukocytes: –	Increase in cell proliferation Chondrogenic phenotype maintenance but decrease over time in micromass pellet cultures Initial enhancement of inflammatory response, followed by its resolution
van Buul [72]	Platelet count: 845.3 × 10^6^/ml Activation: CaCl_2_ Leukocytes: present	Normalization of collagen II, aggrecan, ADAMTS4, MMP13 and PTGS2 expression altered by IL-1ß No influence on GAG content Dose-dependent down-regulation of IL-1ß induced NF-kB activation
Wu [75]	Platelet count: – Activation: Thrombin Leukocytes: –	Dose-dependent increase in chondrocyte proliferation in collagen 3D arthritic model Restoration of collagen II, PG, integrin α1β1 and CD 44 expression inhibited by IL-1ß and TNFα Inhibition of IL-1β, COX-2, and MMP-2 genes expression
Bendinelli [5]	Platelet count: 1,850 ± 320 × 10^6^/ml Activation: Thrombin + CaCl_2_ Leukocytes: present	Antiinflammatory effect: inhibition of NF-kB transactivation activity through HGF, IL4, and TNFα, and inhibition of monocyte-like cells chemotaxis
Spreafico [69]	Platelet count: 1,460 × 10^3^/μl Activation: Ca-gluconate Leukocytes: –	5 % PRPr optimal concentration for chondrocytes proliferation increase Higher PRP concentration does not further induce cell proliferation Increase in collagen II and PG production at day 2 that decreases over time
Drengk [11]	Platelet count: – Activation: CaCl_2_ No leukocytes	Increase in chondrocyte proliferation, but inhibition of chondrogenic markers expression
Pettersson [60]	Platelet count: – Activation: – Leukocytes: –	No beneficial effect on chondrocyte seeded macroporous gelatin microcarriers in terms of histologic characteristics and proteoglycan deposition up to 16 weeks
Saito [61]	Platelet count: 1,081 ± 150 × 10^4^/μl Activation: Thrombin + CaCl_2_ No leukocytes	Increase in GAG content
Akeda [1]	Platelet count: 1,399 ± 174 × 10^3^/ml Activation: Thrombin + CaCl_2_ Leukocytes: –	Stable cell phenotype Increase in cell proliferation and amount of collagen II and PG synthesis, more than PPP or FBS
Gaissmaier [19]	Platelet count: – Activation: Thrombin + Ca–gluconate No leukocytes	Increase in chondrocyte proliferation in dose-dependent manner (stable above 10 %) with inhibition of chondrogenic markers expression in monolayer culture as well as in 3D culture model
Kaps [33]	Platelet count: – Activation: freezing and thawing No leukocytes	Growth promotion activity comparable or superior to mitogenic stimulation by FCS on articular and nasal septal chondrocytes Reduction in ECM formation in chondrocyte/agarose construct
Yang [76]	Platelet count: – Activation: freezing and thawing No leukocytes	Increase in chondrocytes proliferation with 1 % PS Chondrocytes mass formation with 10 % PS Increase in GAG but inhibition of collagen II expression
MSCs + chondrocytes		
Mifune [48]	Platelet count: 230 × 10^4^/ml Activation: Thrombin + CaCl_2_ Leukocytes: –	Promotion of proliferation, adhesion, and migration of MDSCs Increase in cell apoptosis and number of collagen II producing cells
Moreira Teixeira [53]	Platelet count: – Activation: freezing and thawing No leukocytes	High collagen II gene expression and synthesis Chemo-attractant properties in hydrogel Combination with hydrogel allowed retention of PRP at the defect site
Meniscal cells		
Gonzales [23]	Platelet count: 140 ± 20 × 10^9^/l Activation: – Leukocytes: –	Same positive effect as FBS for meniscal cell culture Dose-dependent effect: 10 and 20 % PRP increased proliferation rate and influenced more type I collagen and aggrecan expression at day 7 with respect to 5 % PRP
Ishida [30]	Platelet count: 104.5 × 10^4^/μl Activation: – Leukocytes: –	Increase in meniscal cells proliferation in a dose-dependent manner No effect on collagen I but modulation of GAG synthesis, high biglycan and decorin expression, aggrecan downregulation
Synoviocytes		
Browning [6]	Platelet count: – Activation: – Leukocytes: present	Increase in MMP1, 3, IL-6 and decrease in PDGF-ββ, MIP-1β, RANTES in OA synoviocytes Higher pro-inflammatory response than PPP treatment
Anitua [2]	Platelet count: 494 × 10^6^/ml Activation: CaCl_2_ No leukocytes	Increase in HA secretion, further enhancement in the presence of IL-1ß Angiogenesis switched to a more balanced status No effect on MMP1, 3, and VEGF amounts elicited by IL-1ß
Mesenchymal stem cells		
Hildner [28]	Platelet count: – Activation: – Leukocytes: –	Increase in proliferation Increase in GAG and cartilage markers Better redifferentiation potential than FCS expanded cells
Kruger [42]	Platelet count: 0.6–1.3 × 10^10^/ml Activation: freezing and thawing Leukocytes: <0.3 × 10^4^/ml	Increase CSP migration with 0.1–100 % PRP, especially with 5 % PRP Induction in chondrogenic markers’ expression Induced formation of cartilage matrix rich in PG and collagen II
Moreira Teixeira [53]	Platelet count: – Activation: freezing and thawing No leukocytes	In hydrogel-PL increase in BMSCs proliferation rate, adhesion, and migration No beneficial effect on collagen II mRNA expression in MSCs with chondrogenic medium and PL, but higher expression in control medium and PL
Murphy [55]	Platelet count: 10^6^/μl Activation: CaCl_2_ No leukocytes	PRP is more mitogenic than FBS on MSCs derived from human and rat BM and from rat compact bone Higher increase in MSCs proliferation rate and migration with ucPRP with respect to aPRP
Mishra [52]	Platelet count: 10^6^/ml No activation Leukocytes: present	Induction of MSCs proliferation Increase in chondrogenic markers’ expression (SOX9, Aggrecan)
Drengk [11]	Platelet count: – Activation: CaCl_2_ No leukocytes	Stimulation of BMSCs proliferation and weak chondrogenic differentiation in a 3D environment
Zaky [78]	Platelet count: 1-1.8 × 10^6^/μl Activation: freezing and thawing No leukocytes	Induction of proliferation (more than with FBS and FGF2) during the initial culture passage Induced MSCs chondrogenic differentiation in conditions without FBS
Kakudo [32]	Platelet count: 132.26 × 10^4^/μl Activation: Thrombin + CaCl_2_ Leukocytes: –	Higher increase in ADMSCs proliferation with 5 % PRP Higher proliferation induction with activated PRP versus not activated PRP Decrease in a dose-dependent manner with 10 and 20 % PRP

**Table 2:** In vivo preclinical studies

Publication	Animal model	Lesion type	PRP characteristics	Protocol	PRP effects
Mifune [48]	36 rats	OA	Platelet count: 230 × 10^4^/ml Activation: Thrombin + CaCl_2_ Leukocytes: –	1 injection (30 μl)	Promotion of collagen II synthesis and suppression of chondrocyte apoptosis only when applied with MDSCs at 4 weeks At 12 weeks, lost beneficial effect
Hapa [25]	42 rats	Chondral lesion	Platelet count: 13.8 × 10^9^/l Activation: – Leukocytes: –	1 intra-op injection (150 μl) 1 intra-articular injection (150 μl)	Better cartilage healing and increase in type II collagen expression at 6 weeks
Guner [24]	20 rats	OA	Platelet count: – Activation: Thrombin + CaCl_2_ Leukocytes: –	3-weekly injections (50 μl)	No significant effects regarding cartilage healing at short term (2 weeks after injection cycle)
Serra [66]	36 rabbits	Osteochondral lesion	Platelet count: – Activation: CaCl_2_ No leukocytes	7 injections every 2 days (0.25 ml)	No macroscopic, microscopic, and biomechanical additional benefits from PRP injections up to 19 weeks
Kwon [43]	21 rabbits	OA	Platelet count: 2664 ± 970 × 10^3^/μl Activation: – Leukocytes: –	1 injection (0.3 ml)	Better cartilage regeneration in all OA degrees at 4 weeks, in particular in moderate knee OA
Milano [49]	30 sheep	Chondral lesion	Platelet count: 868 ± 112 × 10^3^/ml No activation No leukocytes	5-weekly injections (3 ml)	Improvement in macroscopic, histologic, and biomechanical cartilage repair after microfractures, with more durable results No hyaline cartilage production up to 12 months
Milano [50]	30 sheep	Chondral lesion	Platelet count: 2 × conc No activation No leukocytes	5-weekly injections (2–3 ml)	Promotion of cartilage healing until 6 months after treatment (not at 12 months) No hyaline cartilage production
Lippross [45]	15 pigs	AR	Platelet count: 1 × 10^6^/μl Activation: – Leukocytes: –	2 injections every 2 weeks (5 ml)	Reduction in IL-6 expression and staining, and VEGF staining Recovery of chondral protein concentration levels Reduction in IL-1ß and IGF-1 on synoviocytes
Milano [51]	15 sheep	Chondral lesion	Platelet count: 1,415 ± 164 × 10^3^/ml Liquid PRP: no activation PRP gel: Ca–gluconate + fibrin glue Leukocytes: –	1 injection (5 ml)	Improvement in macroscopic, histologic and biomechanical scores, no hyaline cartilage production Better results with PRP gel at 6 months
Saito [61]	33 rabbits	OA	Platelet count: 1,081 ± 150 × 10^4^/μl Activation: – No leukocytes	2 injections at 4 weeks and 7 weeks after OA induction (100 μl)	Suppression of OA progression morphologically and histologically by PRP impregnated hydrogel microspheres (not significantly by the use of PRP only)
Carmona [7]	4 horses	OA	Platelet count: 250 ± 71.8 × 10^6^/ml Activation: CaCl_2_ Leukocytes: present	3 injections at 2-week interval (10–20 ml)	Improvement in both degree of lameness and joint effusion, with normal synovial fluid parameters Marked improvement at 2 months maintained up to 8 months

**Table 3:** Clinical studies

Publication	Level of evidence	Pathology	*N* Patients	Protocol	Dose and platelet count	Leukocyte	Activation	Follow-up	Results
Koh [35]	Case series	Knee chondropathy or OA	18 PRP + MSCs	1 injection of PRP + MSCs followed by 2-weekly injections of PRP	3 ml PRP for each injection 5× basal plt count (1.28 × 10^6^ plts/μl)	Yes	Ca-chloride	24 months	Statistical improvement in pain and function
Jang [31]	Case series	Knee chondropathy or OA	65 PRP	1 injection	6 ml PRP platelet count: n.a.	n.a.	No	12 months	Increasing age, and advanced degeneration result in a decreased potential for PRP injection therapy
Hart [26]	Case series	Knee chondromalacia	50 PRP	6-weekly injections After 3 months other 3-weekly injections	6 ml PRP 459,000 plts/μl	n.a.	No	12 months	Significant pain reduction and quality of live improvement in low degree of cartilage degeneration not confirmed by MRI
Patel [58]	Randomized trial	Knee chondropathy or OA	52 Single injections 50 Double injections 46 Saline injections	1 injection versus 2 injections 3 weeks apart	8 ml PRP 310 × 10^3^ plts/μl (238 × 10^7^ plts in total)	No	Ca-chloride	6 months	Significant clinical improvement in PRP group within 2–3 weeks until 6 months, but deteriorating after 6 months No differences between 1 and 2 injections
Gobbi [20]	Case series	Knee chondropathy or OA	50 PRP	2 monthly injections	4 ml PRP 2× basal plt count	Yes	No	12 months	Statistical improvement in pain and function. Good results also in patients with history of cartilage surgery
Koh [34]	Case series	Knee chondropathy or OA	25 PRP/MSCs	1 injection of PRP/MSCs followed by 2-weekly injections of PRP	3 ml PRP for each injection 5× basal plt count 1.28 × 10^6^ plts/μl	Yes	Ca-chloride	17 months	Short-term results revealed reduction in pain and improving function
Torrero [70]	Case series	Knee chondropathy or OA	30 PRP	1 injection	n.a.	No	No	6 months	One PRP injection provided encouraging results in pain and function at 6 months’ follow-up
Napolitano [56]	Case series	Knee chondropathy or OA	27 PRP	3-weekly injections of PRP	5 ml PRP 2.3× basal plt count	n.a.	Ca-gluconate	6 months	PRP proved to be an effective treatment option for OA
Spakova [68]	Comparative trial	Knee chondropathy or OA	60 PRP versus 60 HA	3-weekly injections of PRP	3 ml PRP 4.5× basal plt count	Yes	No	6 months	Superior results in PRP group at short-term evaluation
Sanchez [64]	Randomized trial	Knee chondropathy or OA	79 PRP versus 74 HA	3-weekly injections of PRP	8 ml PRGF platelet count: n.a.	No	Ca-chloride	6 months	Higher percentage of responders in PRP group but no clear superiority of the biological approach
Cerza [8]	Randomized trial	Knee chondropathy or OA	60 ACP versus 60 HA	4-weekly injections of ACP	5.5 ml ACP platelet count: n.a.	No	No	6 months	Superior clinical outcome for PRP in all groups of treatment
Filardo [14]	Randomized trial	Knee chondropathy or OA	55 PRP versus 54 HA	3-weekly injections of PRP	5 ml PRP 5× basal plt count	Yes	Ca-chloride	12 months	Clinical improvement in both groups without significant inter-group difference. Better trend for PRP in low-grade cartilage pathology
Kon [41]	Comparative trial	Knee chondropathy or OA	50 PRP versus 50 LWHA versus 50 HWHA	3-weekly injections of PRP	5 ml PRP 6× basal plt count (6 billion plts in total)	Yes	Ca-chloride	12 months	Best results for PRP in chondropathy group, no statistical difference among treatments for higher degree of cartilage degeneration
Filardo [17]	Comparative trial	Knee chondropathy or OA	72 L-PRP versus 72 L-free-PRP	3-weekly injections of PRP	PRP: 5 ml 949,000 plts/μl PRGF: 5 ml 315,000 plts/μl	PRP: yes PRGF: no	PRP and PRGF: Ca-chloride	12 months	Comparable clinical results with higher post-injective pain in leukocyte-rich PRP group
Kon [13, 36]	Case series	Knee chondropathy or OA	100 PRP	3 injections of PRP 2 weeks apart	5 ml PRP 6× basal plt count (6.8 billion plts in total)	Yes	Ca-chloride	24 months	Significant pain reduction and functional recovery Time-dependent effect of PRP injections with a mean beneficial effect of 9 months
Wang-Saegusa [74]	Case series	Knee chondropathy or OA	261 PRP	3 injections of PRP 2 weeks apart	n.a.	No	Ca-chloride	6 months	Satisfactory results at 6 months’ evaluation in a large cohort of patients
Sampson [62]	Case series	Knee chondropathy or OA	14 PRP	3 injections of PRP 1 month apart	6 ml PRP platelet count: n.a.	n.a.	Thrombin in Ca-chloride suspension	6 months	Clinical improvement at short-term evaluation
Sanchez [63]	Retrospective comparative trial	Knee chondropathy or OA	30 PRP versus 30 HA	3-weekly injections of PRP	6–8 ml PRGF 2× basal plts count	No	Ca-chloride	5 weeks	Better pain control and functional outcome in PRP group
Battaglia [4]	Case series	Hip OA	20 PRP	3-weekly injections of PRP	5 ml PRP platelet count: n.a.	Yes	Ca-chloride	12 months	Clinical improvement but gradual worsening up to 1 year of follow-up
Sanchez [65]	Case series	Hip OA	40 PRP	3-weekly injections of PRP	8 ml PRP platelet count: n.a.	No	Ca-chloride	12 months	Significant pain reduction and functional improvement
Mei-Dan [47]	Quasi-randomized trial	Osteochondral talar lesions	15 PRP versus 15 HA	3 injections of PRP 14 days apart	PRP: 2 ml 2–3× basal plts count	No	Ca-chloride	7 months	Statistically better clinical outcome in PRP group

*Plt* platelet, *n.a.* not assessed, *HA* hyaluronic acid, *L-PRP* leukocyte-rich PRP, *L-free-PRP* leukocyte-free PRP

## Results

**Fig. 1:**
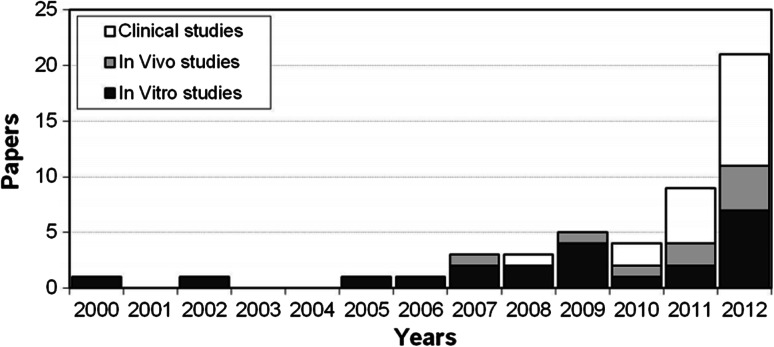
The analysis of per year publication shows the interest in PRP application for the treatment of cartilage lesions or joint degeneration with an increasing number of published studies over time

According to the search strategy, 388 papers were screened, among these 59 met the inclusion criteria: 26 were in vitro, 9 were in vivo, 2 were both in vivo and in vitro, and 22 were clinical studies. The analysis of per year publication showed increasing interest in this topic with an increasing number of published studies over time, in particular with regard to reports documenting results of the clinical injective application of PRP (Fig. 1).

## In vitro studies

### Chondrocytes

Seventeen papers investigated the effect of PRP on chondrocytes (Table 1) [1, 5, 11, 19, 28, 33, 44, 48, 54, 57, 59, 61, 69, 73, 75–77]. In particular, 13 papers reported an increase in chondrocyte proliferation rate. Muraglia et al. [54] even showed that PRP promoted cell proliferation in conditions where fetal calf serum (FCS) had no proliferation stimulating effect, as in chondrocytes from elderly patients. Four papers by Drengk et al. [11], Gaissmaner et al. [19], Kaps et al. [33], and Yang et al. [76] observed, together with the increase in cell proliferation, an inhibition of chondrogenic markers expression. Conversely, 10 papers reported an increase in chondrocyte proliferation rate without affecting chondrogenic phenotype maintenance. Hildner et al. [28] even documented that proliferation and chondrogenic redifferentiation potential were higher when human articular chondrocytes were previously expanded with platelet lysate (PL) instead of FCS. Besides the overall proliferation increase with phenotype maintenance, Park et al. [57] underlined another key point: the time-dependent regulation and the dose-dependency effect. In particular, they tested different PRP concentrations (0.1, 1, 5, 10, and 20 %) showing an increase in cellular viability in a dose-dependent manner. Yang et al. [76] reported that 1 % of platelet supernatant (PS) is sufficient to stimulate chondrocyte proliferation, whereas 10 % PS stimulated chondrocyte mass formation. Spreafico et al. [69] studied PRP releasate (PRPr) at 1, 5, and 10 % and found that 5 % was the optimal concentration to increase chondrocyte proliferation. Moreover, Gaissmaner et al. [19] provided evidence of cell proliferation increase with 1 or 10 % PS, but no further stimulation occurred using concentrations above 10 %.

Together with chondrogenic phenotypic maintenance, other authors also documented an increase in matrix molecule production. Akeda et al. [1] documented that PRP treatment led to higher amounts of collagen II and PG synthesis than platelet poor plasma (PPP) or fetal bovine serum (FBS). Since cell–matrix interactions play an important role in maintaining cartilage homoeostasis, Wu et al. [75] designed a simple 3D chondrocyte model: in a collagen matrix, the authors mimicked an OA environment by IL-1ß and TNFα induction. Also in this model, PRP increased the membrane receptors integrin α1ß1 and CD44 and favoured type II collagen and PG production. In another experimental model, Yin et al. [77] reported that PRP allowed the integration of an agarose construct with cartilage samples, showing a denser extracellular matrix (ECM) deposition in the integration area. Interestingly, Pereira et al. [59] found that the PRP stimulatory effect was limited over time: after an initial positive staining for collagen type II and PG, at 20 doublings the matrix/cells ratio decreased. Similarly, Spreafico et al. [69] documented an increase in PG release 2 days after PRPr treatment, followed by a decrease after 9 days, although at 20 days PG release remained still high.

Four papers focused on the role of PRP in OA chondrocytes as inflammation modulation. Pereira et al. [59] found that PL enhanced the initial inflammatory response and subsequently triggered its resolution through the regulation of nuclear factor kappa B (NF-kB) and cyclooxygenase-2 (COX-2), the principal actors of inflammatory cascade. Van Buul et al. [73] and Bendinelli et al. [5] confirmed the regulation of these key pathways by PRP in inflammatory conditions. Van Buul et al. [73] showed a dose-dependent down-regulation of IL-1ß-induced NF-kB activation, whereas Bendinelli et al. [5] showed that inhibition of NF-kB transactivation activity was mediated by HGF, a cytokine present in PRP α-granules. Moreover, they suggested another anti-inflammatory action by inhibiting monocyte-like cell chemotaxis. Wu et al. [75] also investigated the anti-inflammatory potential of PRP in their 3D system: PRP counteracted the inflammatory cascade elicited by IL-1ß and TNFα, showing an inhibition of IL-1ß, COX-2, and MMP-2 gene expression.

One article investigated the role of PRP as analgesic compound. Lee et al. [44] showed that the addition of PRP to a chondrocyte/hydrogel culture led to an immediate increase in mRNA levels of cannabinoid receptor CB1 and CB2 (receptors involved in analgesic and anti-inflammatory effects).

### Chondrocytes and MSCs co-culture

In a system of OA chondrocytes and muscle-derived MSCs (MDSCs), Mifune et al. [48] observed that PRP promoted proliferation, adhesion, and migration of MDSCs. During chondrogenic pellet culture, PRP tended not only to increase the number of type II collagen-producing cells, but also to increase cell apoptosis, which, however, was not confirmed by the in vivo evaluation. Moreira Teixeira et al. [53] showed high expression and synthesis of collagen II co-culturing chondrocytes and expanded bone marrow MSCs (BMSCs) when PL/hydrogel was added. Moreover, they investigated the retention of PL/hydrogel construct in a cartilage fragment: the combination with hydrogel allowed the retention of PRP at the defect site, filling up irregularities at the cartilage surface.

### Synoviocytes

Anitua et al. [2] investigated the role of PRGF (‘preparation rich in growth factors’: a low-concentrate PRP without leukocytes) on OA synoviocytes with or without exposition to IL-1ß, to mimic the overproduction of proinflammatory cytokines in the joint environment during OA progression. PRGF significantly enhanced HA secretion compared to PPP both with and without IL-1β and switched angiogenesis to a more balanced status, but did not modify the IL-1β-induced rise of matrix metallo-protease (MMP) 1, 3 and vascular endothelial growth factor (VEGF) produced by synovial cells. Indeed, Browning et al. [6] even showed an increase in MMP-1 and MMP-3 in OA synoviocytes incubated with PRP, thus suggesting that the application of PRP to synovial joints might be associated with deleterious effects due a pro-inflammatory response that might lead to an accelerated cartilage catabolism.

### Meniscal cells

Ishida et al. [30] showed the usefulness of PRP not only because of its proliferation effect, but also its induction of GAG synthesis. PRP up-regulated the viability of meniscal cells in a dose-dependent manner, as well as the mRNA expression of biglycan and decorin. Gonzales et al. [23] investigated whether PRP might fully replace FBS for cultured tissue engineering constructs. The study results showed that PRP presents the same positive effect as FBS for meniscal cell culture and showed that dosage is an important aspect of the induced effect: 10 and 20 % PRP increased proliferation rate and influenced more type I collagen and aggrecan expression at day 7 of culture with respect to 5 % PRP.

### Stem cells

Eight papers investigated the effect of PRP on MSCs of different origin: 1 on subchondral cortico-spongious bone (CSP) cells, 1 on commercial human MSCs, 4 on BMSCs, and 2 on adipose-derived MSCs (ADMSCs).

Kruger et al. [42] investigated the migration and chondrogenic differentiation of human subchondral progenitors. In particular, a chemotactic assay revealed that PRP significantly stimulated the migration of CSPs, together with their chondrogenic differentiation and production of PG and collagen type II. Zaky et al. [78] and Drengk et al. [11] confirmed an induced chondrogenic differentiation of BMSCs, which also presented a higher proliferation rate. Mishra et al. [52] documented the same behaviour on MSCs with a higher proliferation rate and a selective differentiation along the chondrogenic line: SOX9 and aggrecan (chondrogenic markers) were increased much more than RUNX2 (osteogenic marker). Conversely, Moreira Teixeira et al. [53] reported that PL, besides inducing a significant increase in BMSCs proliferation rate and migration, did not induce an increase in collagen type II.

Hildner et al. [28] focused on ADMSCs and showed strongly enhanced proliferation rates with retained chondrogenic differentiation potential and even a tendency toward increased chondrogenic differentiation of PL-expanded ADMSCs compared to FCS. Kakudo et al. [32] studied the proliferation of ADMSCs treated with PRP with or without activation and at different concentrations (1, 5, 10, or 20 %). Results showed the importance of both PRP activation and correct dosage: in fact, the stronger promotion of proliferation was observed in PRP activated with calcium chloride and autologous thrombin and applied at 5 %, whereas at higher platelet concentrations the proliferation rate decreased in a dose-dependent manner.

Finally, Murphy et al. [55] tested two different types of PRP: one derived from human adult peripheral blood and one derived from human umbilical cord blood (ucPRP), showing the superiority of ucPRP with regard to MSCs proliferation and migration induction.

## In vivo preclinical studies

Concerning in vivo preclinical studies dealing with PRP injective treatment, we found 11 papers: 3 on rat, 3 on rabbit, 3 on sheep, 1 on pig, and 1 on horse, which showed heterogeneous results for heterogeneous indications.

Five papers focused on OA treatment. Contrasting results have been reported in the small animal model. In fact, whereas Guner et al. [24] did not find any immediate (2 weeks after the injection cycle) benefit of PRP on cartilage tissue in rat joints previously damaged with intra-articular formalin injection, Mifune et al. [48] found in a rat OA model, induced by monosodium iodoacetate injection, that PRP had no marked effect by itself, but increased the cartilage repair effect of MDSCs, with a better histologic appearance, higher number of cells producing type II collagen, and lower levels of chondrocyte apoptosis at 4 weeks, although at 12 weeks its effects were lost. Kwon et al. [43] confirmed the benefit of PRP in a rabbit model of collagenase-induced OA: intra-articular injections influenced positively cartilage regeneration in all OA severity degrees, with a more evident effect in moderate OA. Saito et al. [61] used a rabbit OA model of anterior cruciate ligament resection for the treatment with gelatin hydrogel microspheres impregnated with PRP: injections markedly suppressed OA progression both morphologically and histologically (less significant results were obtained by the use of PRP only). Finally, Carmona et al. [7] used a large animal model to analyze the effect of PRP injections: in a study on 4 horses with OA, 3 injections of PRP led to a significant improvement in both the degree of lameness and joint effusion. The most marked improvement was observed 2 months after treatment and persisted for 8 months with no adverse events.

Five studies focused on the injective treatment of chondral or osteochondral lesions. Also in this case, results were controversial. Serra et al. [66] performed 7 PRP injections every other day in rabbit joints where a full-thickness osteochondral lesion was previously made surgically on the medial femoral condyle. A fibrous–cartilaginous tissue was found with no benefit from PRP. Hapa et al. [25] evaluated PRP as augmentation in rat cartilage lesions after microfractures: at week 6, the microfracture group score was worse than that of the PRP + microfracture group, which had an increased degree of type II collagen staining. Milano et al. [51] used one PRP injection as augmentation procedure of microfracture in a sheep model. Although no hyaline cartilage was obtained, PRP offered better macroscopic, histologic, and biomechanical results. The PRP administration modality proved to be important for the final outcome, with better results when PRP was surgically applied as a gel over the treated lesion. However, this required a more invasive approach. Thus, in a further evaluation in sheep, Milano et al. [49, 50] focused on the injective approach: 5-weekly injections of PRP promoted a better spontaneous repair and also a better and more durable reparative response when applied after microfractures with respect to isolated microfractures, albeit without producing hyaline cartilage.

Finally, only 1 paper focused on rheumatoid arthritis (RA). Lippross et al. [45] reproduced RA in pigs: the animals were systemically immunized by bovine serum albumin (BSA) injections, and arthritis was induced by intra-articular BSA injection. The injection of PRP attenuated the arthritic changes on synovium and cartilage by modulating the activity of inflammation mediators. In particular, IL-6 and VEGF staining was reduced, but concerning gene expression, only IL-6 levels were significantly lower after PRP application. Focusing on protein quantification, all chondral protein concentrations returned to healthy tissue levels, and in synovial samples, besides the low levels of IL-6 and VEGF, the authors showed a reduction in IGF-1 and IL-1 in PRP groups, whereas TNFα was not altered.

## Clinical studies

Intra-articular clinical application of PRP has been tested in several clinical studies to date. The present search identified 22 clinical trials that met the inclusion criteria: among these, 13 were case series, 4 were comparative studies, and 5 were randomized trials. The majority of the available papers deal with application in the knee.

The first comparative evaluation was performed by Sanchez et al. [63] in 2008 who published a retrospective observational study on 60 patients, 30 treated with 3 knee intra-articular injections of PRGF and 30 with 3 injections of hyaluronic acid (HA). Results at 5 weeks were encouraging, with PRGF showing better efficacy in pain control. Afterwards, Kon et al. [41] in 2011 performed a prospective comparative study testing PRP against low molecular weight HA (LW–HA) and high molecular weight HA (HW–HA) in 3 homogeneous groups of 50 patients each. The results showed a better performance for the PRP group at 6 months of follow-up. In particular, PRP produced superior results in the ‘chondropathy’ group. Conversely, in the early OA group the difference with HA was not significant and in the severe OA group no difference in clinical outcome was observed. Another interesting finding was that patients aged up to 50 years old had a greater chance to benefit from the PRP approach. The same authors were the only ones to compare two different PRP preparations: high-concentrate leukocyte-rich PRP versus low-concentrate leukocyte-free PRP. One hundred forty-four patients were treated and evaluated up to 12 months and comparable positive results were obtained with both treatments, with the only difference being that the PRP-leukocyte group suffered from more swelling and pain reaction immediately after the injections [37]. Spakova et al. [68] also compared the efficacy of PRP versus visco supplementation in 120 patients. An increase in the clinical scores was reported in both groups at 6 months, but statistically superior results were found in the PRP group.

Recently, five randomized controlled trials have been published. Sanchez et al. [64] investigated the efficacy of single-spinning leukocyte-free PRP compared to HA in 153 patients evaluated up 6 months of follow-up. The only aspect where a clear superiority of PRP was found was the percentage of responders (patients with at least 50 % of pain reduction), which was significantly higher in the PRP group. Besides this finding, the study did not show that PRP in moderate/severe OA was more effective than HA. Similar considerations were made by Filardo et al. [14], according to the preliminary results (109 patients) of their randomized double-blind trial comparing PRP and HA: no statistical inter-group difference was reported and just a tendency toward better results for the PRP group at 6 and 12 months of follow-up was found in patients affected by low-grade cartilage degeneration (Kellgren Lawrence up to 2). Conversely, Cerza et al. [8] treated 120 patients by either autologous conditioned plasma (ACP, a low-concentrate PRP without leukocytes) or HA. Surprisingly, the ACP group showed a significantly better performance than HA in all groups of treatment, including patients affected by grade 3 knee OA. Furthermore, the clinical gap between treatments increased over time in favour of ACP. Finally, a recent randomized trial by Patel et al. [58] was the first to test PRP versus saline. Seventy-eight patients affected by Kellgren grade I–III OA were included and treated bilaterally with one injection of PRP, two injection of PRP (3 weeks apart) or one injection of saline. Despite the low number of patients included [12], a significant difference was observed between PRP and saline solution in terms of clinical outcome. Interestingly, no difference was reported among patients who received one or two PRP injections.

Only one paper investigated the efficacy of PRP versus HA in osteochondral talar lesions on 30 patients [47]. In the short-term 28-week evaluation a superior clinical performance was found in the PRP group.

## Discussion

This systematic review confirmed the increasing interest in PRP as an injective treatment for cartilage degeneration and OA, with an increasing number of published studies over time.

PRP is a fashionable treatment, offering the possibility to deliver a high concentration of autologous growth factors and bioactive molecules in physiologic proportions, with low costs and in a minimally invasive way. This explains the wide application of this blood derivative to several tissues and heterogeneous pathologies in different fields of medicine [38]. The rationale for using platelets for the treatment of different tissues is that they constitute a reservoir of growth factors that are critical to regulate the tissue healing process, which is quite similar in all kinds of tissues. However, whereas the rationale for PRP use in other tissues is clear, since platelets represent the first response to a tissue damage where they participate in stopping the vessel bleeding and trigger the healing cascade [9], less intuitive is the rationale for PRP use in cartilage, which is a physiologically vessel-free tissue. Moreover, whereas some molecules such as TGF-ß might justify its use in cartilage, PRP also contains other molecules such as VEGF that do not take part or might even jeopardize cartilage homeostasis and regeneration [48, 72]. Thus, it is mandatory to investigate whether the overall effect of PRP is also beneficial for the peculiar requirements of cartilage tissue before an indiscriminate human application.

The systematic analysis of in vitro studies published up to now shows an overall positive effect of PRP on cartilage tissue. Besides some controversial results, most of the findings supported the role of PRP in increasing chondrocyte proliferation, without affecting chondrogenic phenotype and with an increase in the production of matrix molecules. These properties of PRP have provided positive results also in the animal model: preclinical studies confirmed the usefulness of PRP treatment in different pathology models, with good results in cartilage regeneration after acute focal lesions, as well as in the more complex environment of joint osteoarthritic degeneration, and even in the challenging RA setting.

Clinical studies on PRP injective treatment for joint degeneration also showed overall good results. Nonetheless, both the rapid clinical benefit and the limited effect over time are in contrast with the timing required by a hypothetically induced cartilage regeneration process. Despite the wide majority of studies focusing on cartilage tissue, it is actually likely that the clinical benefit reported after PRP injection is attributable to other action mechanisms.

An intra-articular injection does not just target cartilage, instead PRP might influence the entire joint environment, and some in vitro studies confirm the effects of PRP on other cell sources. Synoviocytes are affected by platelet releasate, as well as meniscal cells and also MSCs that seem to be induced by PRP and act synergically toward tissue healing. The chemo-attractant activity of PRP may contribute to the recruitment of other cells that might migrate into the damaged tissues, thus triggering the healing response [42, 53]. PRP has several potential effects by enhancing the cell signalling cascade in all joint tissues and inducing positive changes in the whole joint environment through a milieu of actions. Among these, tissue regeneration is actually not the only and maybe not the most important PRP mechanism of action, and increasing evidence supports the complex role of PRP in modulating inflammation. PRP showed both pro- and anti-inflammatory activities: an initial pro-inflammatory action [59] was reported, with synoviocyte stimulation for MMP and cytokine release [2], followed by a limitation of the inflammatory response by decreasing inflammatory molecules and preventing chemotaxis of monocytes-like cells [44, 75].

An overall down-modulation of the joint inflammation can explain the well-documented pain reduction, which is the most prominent and disabling symptom of cartilage lesions and knee OA. However, some findings suggest another intriguing aspect of PRP action mechanism, with a direct analgesic effect: Lee et al. [44] showed the role of PRP in the augmentation of cannabinoid receptors CB1 and CB2, which might be involved in the analgesic effects. Further studies need to focus on understanding and possibly optimizing the analgesic and anti-inflammatory effects of PRP.

PRP might not lead to hyaline cartilage regeneration and might not change the clinical history with significant disease-modifying properties, but it still might offer a clinical benefit with symptoms and function improvement and possibly a slowdown of the degenerative processes.

The central feature in OA cartilage degeneration is the so-called apoptosis (programmed cell death); thus, chondrocytes apoptosis is a potential therapeutic target for OA interventions. The exact mechanism behind the PRP regulation of the apoptotic pathway is unclear, but it is likely that PRP might have an overall effect in slowing down the apoptosis cascade. Among the hypothesized mechanisms, recent findings identified IGF-1 protein as a possible effector of apoptosis inhibition: Yin et al. [77] found that IGF-1 may down-regulate the expression of programmed cell death 5 (PDCD5), thus inhibiting the apoptosis of osteoarthritic chondrocytes. Interestingly, Mifune et al. [48] observed an increased cell apoptosis in the in vitro setting, which, however, was not confirmed by the subsequent in vivo experiment, where lower levels of apoptosis were detected. Thus, the authors suggested that it was the complex interaction of PRP with the different joint structures (synovium, fat pad, bone marrow,…), which might positively influence chondrocytes apoptosis.

The controversial findings reported underline the limits of preclinical studies, which do not exactly represent the peculiar human pathophysiology. Nonetheless, although such experimental settings do not replace the fundamental role of robust clinical trials, in vitro studies can suggest mechanisms of action and directions for improvement and might explain some controversial findings in the reports of PRP application in humans. As for other tissues [71], in vitro studies have shown the importance of the dosage of the potent platelet-derived growth factors, with different platelet concentrations leading to different results. Activation might also play an important role, as well as the appropriate cell population which is also a key aspect for obtaining optimal results [38]. With regard to this, leukocytes are a controversial PRP component, since some authors attribute better results to leukocyte depletion, because of the supposed deleterious effects of proteases and reactive oxygen species released from white cells, whereas other authors consider them as a source of cytokines and enzymes that may also be important for the prevention of infections [17]. Several other variables have to be considered, such as the preparation methods and the consequent presence of other cells, storage modalities, application protocols, and many other aspects that might not be of secondary importance for determining PRP properties and clinical efficacy [38]. The number of names and acronyms encountered searching for studies on this biological treatment approach, such as PRP, PRGF, ACP, PL, clearly represents the complexity of this field and explains the difficulties in literature analysis, study comparison, and understanding some contradictory results.

With the limits of a complex field still in its infancy, few studies and some controversial results, this systematic review still showed some important aspects. The first one is that the increasing interest in this topic is being translated into research with a growing number of papers published over time, which show promise in shedding some more light on PRP use in the near future. The second one is that, besides the limits and sometimes controversial findings of in vitro and animal studies, the preclinical literature documented an overall support toward PRP application for the injective treatment of cartilage lesions and OA. Moreover, some conclusions can be drawn also with regard to human application, which can be of clinical usefulness. The first one is the safety of PRP injections, with no major adverse events reported in the literature and only some reports of self-limiting immediate pain and swelling reaction [17, 36, 41]. The second one is that all studies seem to agree on an overall clinical benefit of PRP. Better results with respect to saline have been shown, and some studies suggest a slight superiority of PRP with respect to visco supplementation [8, 14, 58, 64]. However, not all patient categories present the same results that are more significant in younger patients affected by not too advanced degeneration, and the clinical benefit is limited over time and can roughly be estimated at less than 1 year [13]. This might suggest that this treatment could be applied in cycles to ensure longer lasting results and postpone more invasive procedures. Finally, another aspect emerges from the literature analysis: whereas among the available techniques none clearly seemed to offer superior clinical results [17], it appears clear that there is room for a better targeting of this PRP application. Several aspects still need to be studied to understand the mechanism of action of PRP and give better treatment indications and possibly to optimize the procedure and improve the potential of this biological minimally invasive approach for the treatment of cartilage degeneration and OA.

## Conclusions

One of the emerging fields of PRP treatment is its injective application for cartilage degeneration and OA, as shown by an increasing number of papers published on this topic over time. Preclinical evidence supports the use of PRP injections that might promote a favourable environment for joint tissues healing, targeting not only cartilage but also synovial and meniscal tissues. A few high-quality trials have been published, which showed the clinical usefulness of PRP but only with an improvement limited over time and mainly in younger patients not affected by advanced degeneration. Many biological variables might influence the clinical outcome and have to be studied to optimize PRP injective treatment in case of cartilage degeneration and OA.
